# Supplementary material for: Electrification at water–hydrophobe interfaces
Source: Nat Commun. 2020 Oct 20;11:5285. doi: 10.1038/s41467-020-19054-8 (PMC7576844; doi:10.1038/s41467-020-19054-8)
Supplement: Supplementary file 1 — Supplementary Information [file 41467_2020_19054_MOESM1_ESM.pdf]

Supplementary Information

Electrification at Water-Hydrophobe Interfaces

Nauruzbayeva et al.

Supplementary Section 1: Atomic force microscopy

A Veeco Dimension Icon Scanning Probe Microscope (SPM) was used to quantify the topographies of our samples: (1) flat borosilicate glass slides covered with FDTS (Figure S1A) and (2) inner surfaces of polypropylene micropipette tip (Figure S1B). We analyzed several spots of 50×50 μm^2 area on each sample.

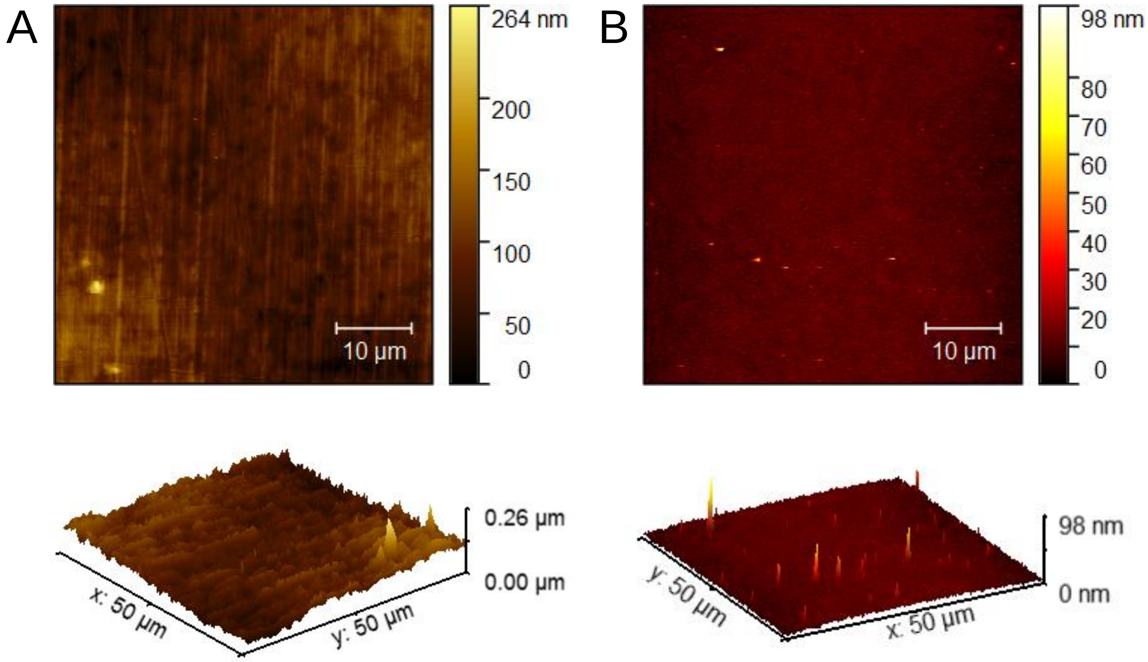

Supplementary Figure 1. Atomic force microscopy images of (A) a borosilicate glass covered with FDTS, and (B) the inner surface of polypropylene tip.

Supplementary Section 2: Experimental setup

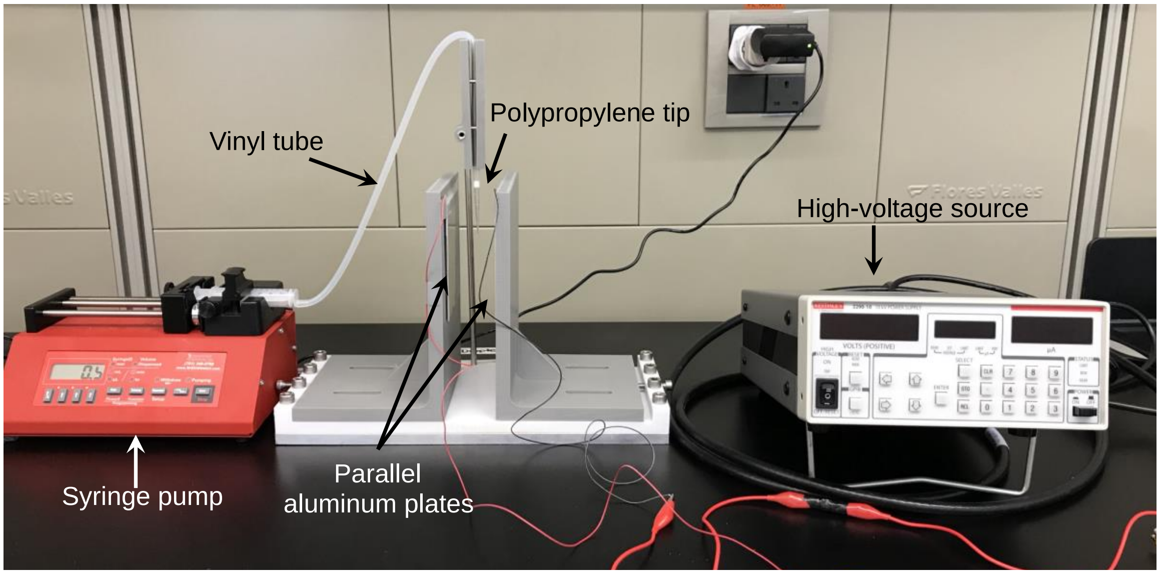

Supplementary Figure 2. Experimental set-up for investigating the behaviors of aqueous (pendant) droplets formed using hydrophobic and hydrophilic capillaries inside a capacitor. The capacitor facilitated uniform electric field strengths. Once the droplet was formed, voltage was applied to the capacitor plates, and it was gradually increased until the pendant droplet detached from the tip. Images were recorded using a high-speed camera and analyzed for further analyzed.

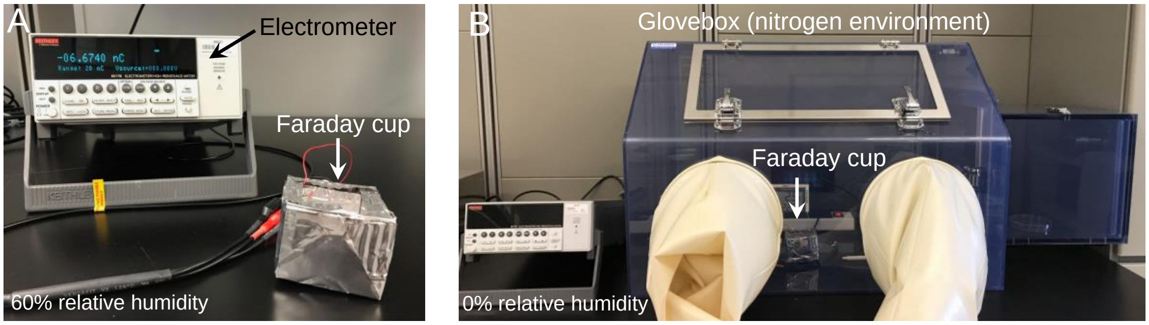

Supplementary Figure 3. Experimental set-up for measuring the electrical charge of pendant droplets by dispensing them into a Faraday cup connected to the Electrometer in (A) laboratory conditions and in (B) nitrogen environment.

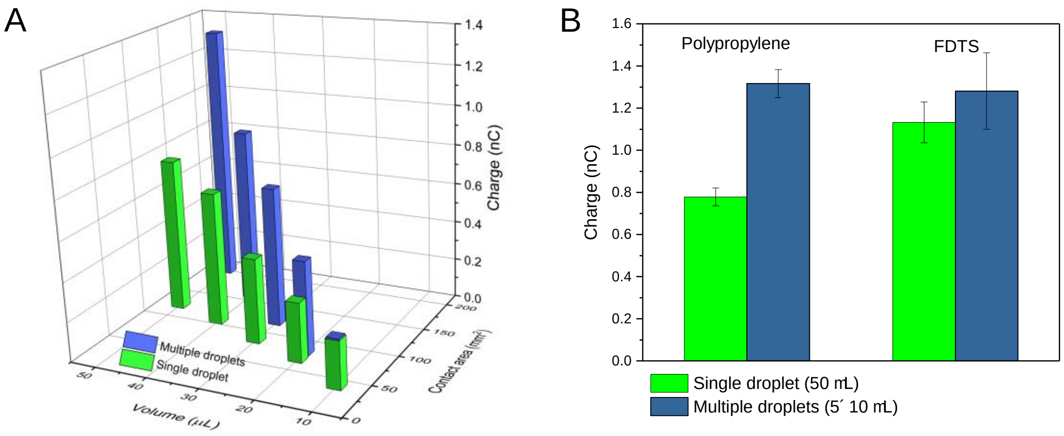

Supplementary Figure 4. (A) Correlation between the electrical charges carried by water droplets dispensed from polypropylene capillaries/pipettes and the contact area between water and tip's surface. To study the effect of the liquid-solid interfacial area on the electrification, we compared the electrical charges of 20-50 μL water droplets (green bars) with those of two-to-five 10 μL droplets (blue bars) using the electrometer set up. (B) Another elucidation of the effect of liquid-solid interfacial area on the electrification by dispensing 50 μL of deionized water in five aliquots of 10 μL from cylindrical FDTS-coated glass capillaries and comparing it against conical polypropylene pipettes. The differences are statistically significant only for the conical capillaries; for cylindrical capillaries the liquid-solid interfacial area is the same for 50 μL and 5×10 μL drops. Error bars represent the standard deviation of ten measurements.

Supplementary Section 3: Investigation of the roles of water-polypropylene and water-air interactions

We compared the extents of electrification when a volume V_1 of water was dispensed from conical polypropylene capillaries by (i) dispensing the entire volume in a single unloading, or (ii) dispensing n portions of volume $V_2 = 10$ μL, such that $n \times V_2 = V_1$. Here, the liquid-solid area was fixed, but the air-water interfacial area was larger in the second case. We found that the average electrical charges in both the scenarios were similar. Therefore, we considered the contribution of the air-water interface on the electrification to be significantly lower than the water-polypropylene interface.

Supplementary Table 1. The comparison of the charges of water droplets after dispensing from polypropylene tip either discharged at once or dispensed in smaller portions.

Volume of water (μL)	Charge of the water droplet dispensed at once (nC)	Charge of the water dispensed in portions (nC)
**20**	0.30±0.01	0.32±0.13
**30**	0.43±0.02	0.47±0.15

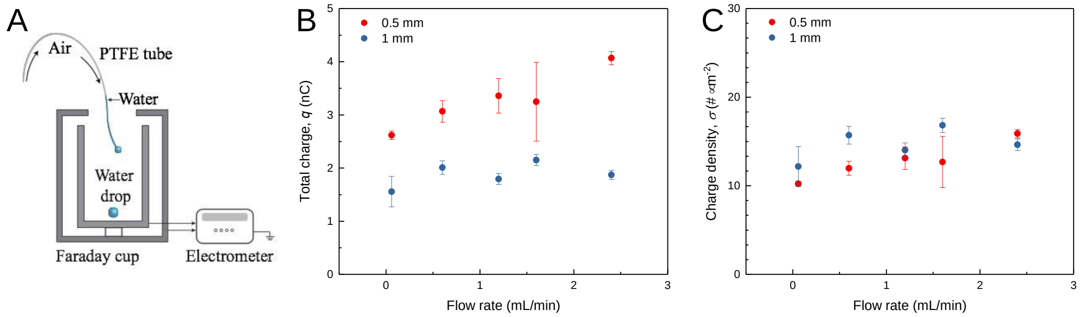

Supplementary Figure 5. (A) Schematics of experimental set-up: a known volume of water was withdrawn using a PTFE tube and subsequently pushed out by a column of air. Charges carried by water drops were measured by a Faraday cup connected to an electrometer. (B) Correlation between the total electrical charge carried by 200 µL water dispensed from PTFE tubes of inner diameters of 0.5 mm (red dots) and 1 mm (blue dots) as a function of the rate of dispensing (controlled by a syringe pump). The larger liquid-solid contact area in the tube of 0.5 mm diameter led to higher electrical charging in comparison with the tube with 1 mm diameter. (C) When the total charge in either scenario is normalized by the liquid-solid interfacial area, similar charge density is obtained. This is expected because the material composition of the tubes is the same. Error bars in each panel represent the standard deviation of five measurements.

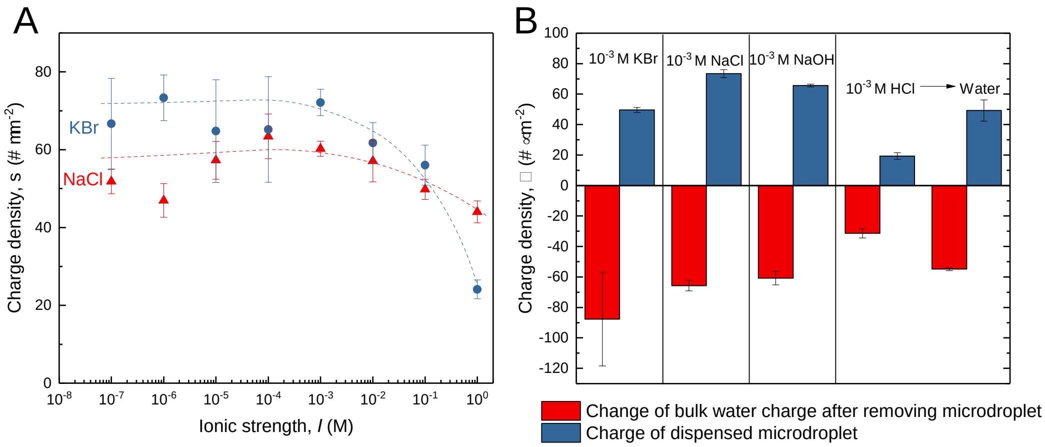

Supplementary Figure 6. (A) Effects of ionic strength of KBr and NaCl solutions in the range 10^{-7}-1 M on the electrification at water-hydrophobe interfaces. The solutions were drawn into polypropylene pipettes from charge-neutral reservoirs and dispensed into a Faraday cage connected to an electrometer to quantify the electrical charge. (Dotted lines have been added to guide the eye). (B) Using an electrometer, we quantified the electrical charges of tiny aliquots (50 µL) from aqueous reservoirs (1 mL) whose ionic strength was adjusted by KBr, NaCl, NaOH, and HCl, using polypropylene pipettes. The electrical charges of the reservoirs were after the withdrawals (red) were always equal and opposite to those of the aliquots (blue). The magnitude of the electrification was quite similar for KBr (1mM), NaCl (1mM), and NaOH (pH 11), but it was significantly lower for HCl (pH 3). After the experiments with pH 3 solutions, when we used the same pipettes for water, the electrification corresponded to the original surface charge density of polypropylene. These results demonstrate that acids do not necessarily "neutralize" the surface charge. (Note: the surface charge densities were obtained by normalizing the observed charges by the solid-liquid interfacial areas inside the pipettes prior to dispensing.) Error bars in each panel represent the standard deviation of ten measurements.

Supplementary Section 4:

Derivation of an equation relating the dependence of the tilting angles, α, of pendant water droplets on the excess charges, q, carried by them and the applied electric field strengths, *E*, through a variational analysis

We estimated the excess charge of the droplets (q) as:

$$q = \frac{mg \tan \alpha}{E} \qquad [1]$$

where m is the mass of the drop, g is the acceleration due to gravity, α is a tilting angle, and *E* is the electric field inside the capacitor.

$$\tan \alpha = \frac{qE}{mg} \qquad [2]$$

If we express this expression in logarithmic form, we get:

$$\log(\tan \alpha) = \log(q) + \log(E) - \log(mg) \qquad [3]$$

This equation on differentiation gives,

$$\frac{\sec^2 \alpha \times \Delta\alpha}{\tan \alpha} = \frac{\Delta q}{q} + \frac{\Delta E}{E} \qquad [4]$$

where $\Delta\alpha$ is the change in the tilting angle as the electric field is changed by ΔE and Δq refers to the ion-exchange between the drop and the water reservoir above (Supplementary Figure 2, Figures 2A and 2C). Note, that derivatives of constant terms (weight and liquid-solid interfacial area) were zero. Equation [4] on minor reorganization yields,

$$\frac{\Delta q}{q} = \left(\frac{2\Delta\alpha}{\sin 2\alpha}\right) - \frac{\Delta E}{E} \qquad [5]$$

(i) In the absence of a water reservoir, Equation [5] reduces to:

$$\Delta\alpha = \frac{\Delta E}{E} \times \left[\frac{\sin 2\alpha}{2}\right] \qquad [6]$$

(ii) In the presence of a water reservoir, Equation [5] remains as:

$$\Delta\alpha = \left[\frac{\Delta E}{E} + \frac{\Delta q}{q}\right] \times \left[\frac{\sin 2\alpha}{2}\right] \qquad [7]$$

Connecting excess charge density of pendant water droplets to the excess charges and the applied electric field strengths through variational analysis:

$$\sigma = q/A_o = mg\tan\alpha/EA_o \qquad [8]$$

Differentiating Equation [8],

$$\Delta\sigma = \frac{\Delta q}{A_o} \qquad [9]$$

From Equations [8 & 9],

$$\frac{\Delta\sigma}{\sigma} = \frac{\Delta q}{q} \qquad [10]$$

This equation shows that the changes in the excess charge density, $\Delta\sigma$, depend only on the changes in the excess charge carried by the droplets, Δq, and not on the applied electric field strength.

An alternative Derivation of Equation [10]:

$$\sigma = q/A_o = mg\tan\alpha/EA_o \qquad [8]$$

Following the same treatment as in the derivation above, let's apply logarithm on either side to Equation [8]:

$$\log(\sigma) = \log(mg) + \log(\tan\alpha) - \log(E) - \log(A_o) \qquad [11]$$

Differentiating Equation [11],

$$\frac{\Delta\sigma}{\sigma} = \frac{\sec^2\alpha\times\Delta\alpha}{\tan\alpha} - \frac{\Delta E}{E} \qquad [12]$$

Using Equations [4] and [12], we get

$$\frac{\Delta\sigma}{\sigma} = \left(\frac{\Delta q}{q} + \frac{\Delta E}{E}\right) - \frac{\Delta E}{E} \qquad [13]$$

which yields,

$$\frac{\Delta\sigma}{\sigma} = \frac{\Delta q}{q} \qquad [14]$$

This result is same as derived in Equation [10].

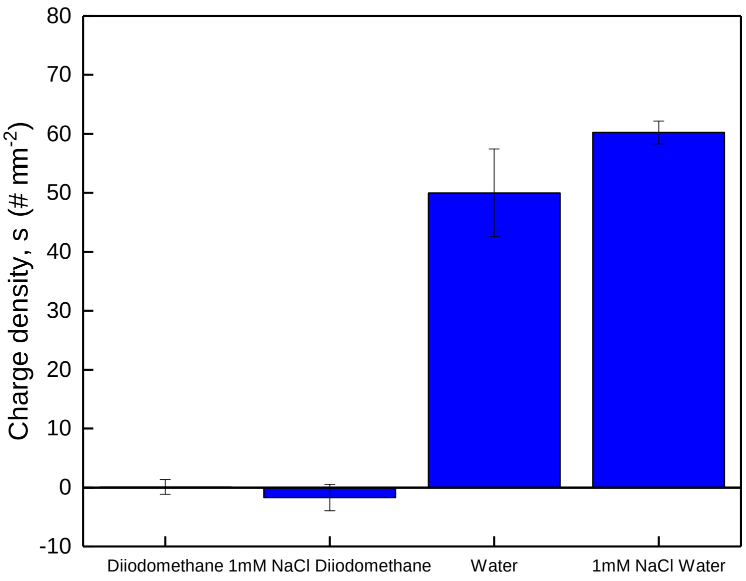

Supplementary Figure 7. Electrical charges of single drops of diiodomethane, diiodomethane containing 1mM NaCl, water, and water containing 1mM NaCl, manually dispensed from polypropylene capillaries at the rate of ~3 mL min^{-1}. Due to the low dielectric constant of diiodomethane (ε_r = 5.3), even the addition of 1 mM salt did not enhance its electrification during pipetting. In contrast, significantly higher electrification was observed at the water-polypropylene interface due to water's intrinsic ions and higher dielectric constant (ε_r = 80). Addition of 1mM NaCl increased the electrification for water. Error bars represent the standard deviation of five measurements.

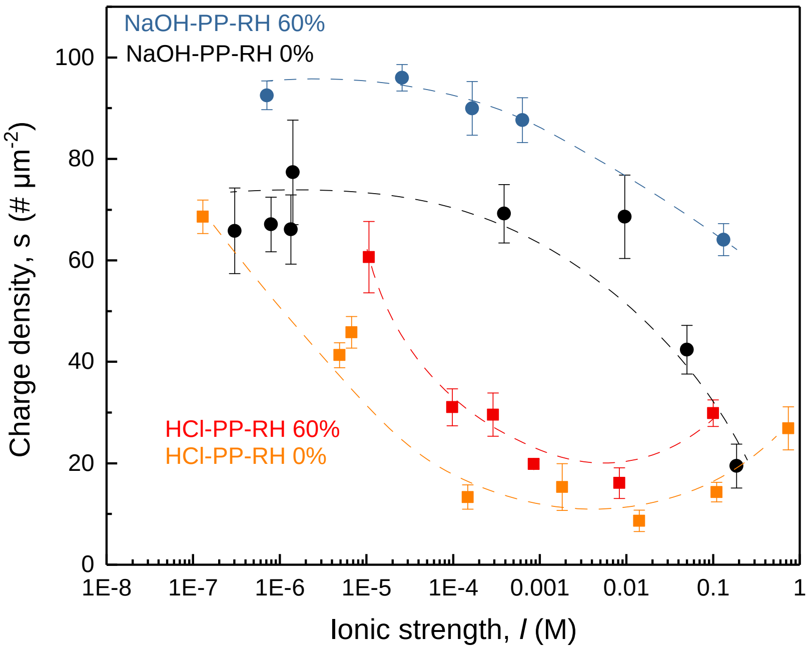

Supplementary Figure 8. The surface charge density of aqueous solutions, ionic strengths adjusted by NaOH (dots) and HCl (squares), dispensed from polypropylene (PP) tips (air-filled scenario). The measured in ~0% and ~60% relative humidity (RH) environments by the Faraday cup method. (Dotted lines have been added to guide the eye.) Error bars represent the standard deviation of ten measurements.

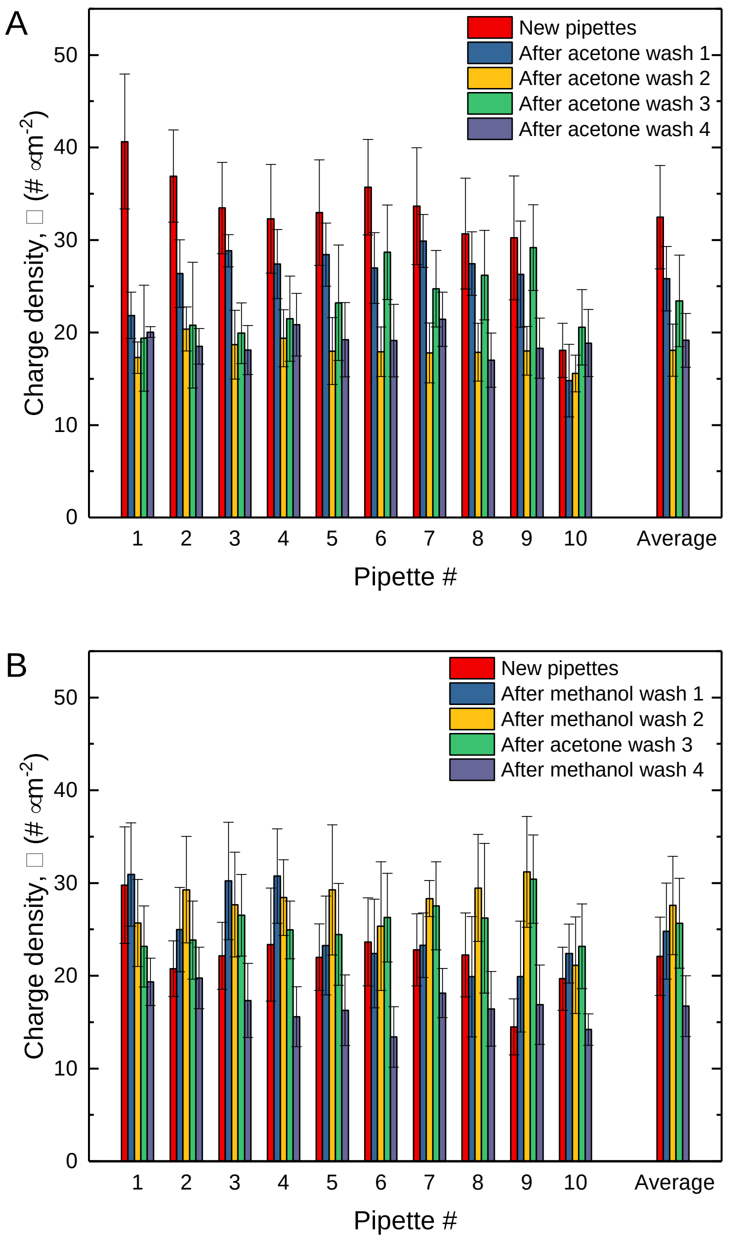

Supplementary Figure 9. Electrical charges of water droplets manually dispensed at the rate of ~3 mL min^{-1} from new polypropylene capillaries (red) and after the capillaries were washed with (A) acetone or (B) methanol and dried overnight. We performed the cycle of washing with a solvent, drying and dispensing water droplets four times for each pipette. The last set of bars represents the average results of performed measurements. The charges were measured by a Faraday cup connected to an electrometer. Error bars in each panel represent the standard deviation of ten measurements.

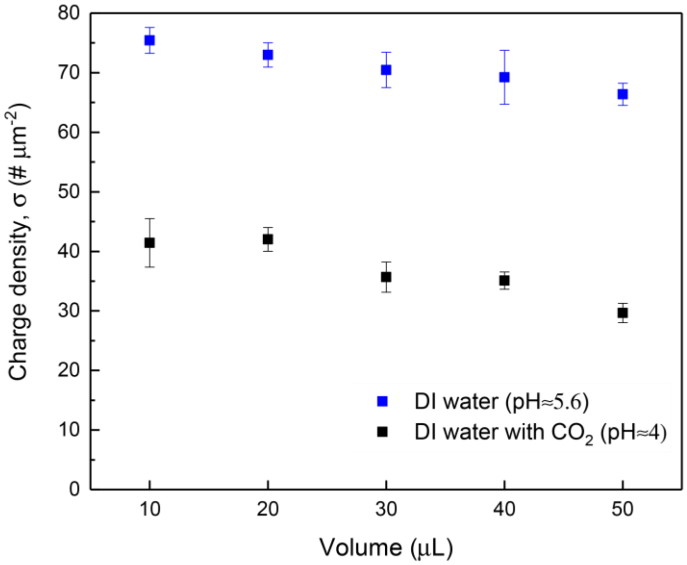

Supplementary Figure 10. The surface charge density of deionized water (blue dots) and water bubbled with CO_2 (black dots) exiting polypropylene tip. We reached pH ≈ 4 by supersaturating water with CO_2 gas. The charges were measured by the Faraday cup method at nitrogen environment. Error bars represent the standard deviation of ten measurements.
