# Supplementary material for: Electrification at water–hydrophobe interfaces
Source: Nat Commun. 2020 Oct 20;11:5285. doi: 10.1038/s41467-020-19054-8 (PMC7576844; doi:10.1038/s41467-020-19054-8)
Supplement: Supplementary file 3 — Description of Additional Supplementary Files [file 41467_2020_19054_MOESM3_ESM.docx]

**Description of Additional Supplementary Files**

**Electrification at Water-Hydrophobe Interfaces**

Nauruzbayeva et al.

**File name: Supplementary Movie 1.**

**Description:** High speed imaging of tilting and detachment of a pendant water droplet dispensed from a polypropylene capillary (water-filled case) inside a parallel plate capacitor. The drop tilted towards the positively charged plate of the capacitor. (Distance between the plates was 4 cm; voltage applied to the positively charged plate: 8 kV).

**File name: Supplementary Movie 2.**

**Description:** High speed imaging of tilting and detachment of a pendant water droplet dispensed from a polypropylene capillary (air-filled case) inside a parallel plate capacitor. The drop tilted away (i.e., repelled) from the positively charged plate of the capacitor. (Distance between the plates was 1.5 cm; voltage applied to the positively charged plate: 9 kV).

**File name: Supplementary Movie 3.**

**Description:** Tilting of sequential 10 μL water droplets dispensed from a partially-filled polypropylene capillary (initial volume: 200 μL) inside a parallel plate capacitor. The first few drops tilted towards the positively charged plate, the following were electroneutral, and the rest repelled from the positively charged plate. (Distance between the plates of the capacitor: 3 cm; voltage cm; voltage applied to the positively charged plate: 5 kV).

**File name: Supplementary Movie 4.**

**Description:** Tilting of a pendant water droplet dispensed from a polypropylene capillary (air-filled) in a parallel plate capacitor. The droplet tilted away from the positively charged plate. (Distance between the plates of the capacitor: 3 cm; cm; voltage applied to the positively charged plate: 1-2.5 kV).

**File name: Supplementary Movie 5.**

**Description:** Tilting of a pendant water droplet dispensed from a FDTS-coated glass capillary (air-filled) inside a parallel plate capacitor. The droplet tilted away from the positively charged plate. (Distance between the plates of the capacitor: 3 cm; voltage applied to the positively charged plate: 1-3 kV).

**File name: Supplementary Movie 6.**

**Description:** Tilting of a pendant water droplet dispensed from a borosilicate glass capillary (air-filled) inside a parallel plate capacitor. The drop tilted towards the positively charged plate. (Distance between the plates of the capacitor: 3 cm; voltage applied to the positively charged plate: 1-5 kV).

**File name: Supplementary Movie 7.**

**Description:** Tilting of a pendant water droplet dispensed from a polypropylene capillary (water-filled) inside a parallel plate capacitor. The drop tilted towards the positively charged plate. (Distance between the plates of the capacitor: 3 cm; voltage applied to the positively charged plate: 1-4 kV).

**File name: Supplementary Movie 8.**

**Description:** Tilting of a pendant water droplet dispensed from a borosilicate capillary (water-filled) inside a parallel plate capacitor. The drop tilted towards the positively charged plate. (Distance between the plates of the capacitor: 3 cm; voltage applied to the positively charged plate: 1-5 kV).

**File name: Supplementary Movie 9.**

**Description:** Tilting of a pendant methanol droplet dispensed from a polypropylene capillary (air-filled) inside a parallel plate capacitor. No tilting observed in the applied voltage range. (Distance between the plates of the capacitor: 2 cm; range of the applied voltage: 1-5 kV).

**File name: Supplementary Movie 10.**

**Description:** Tilting of a pendant hexadecane droplet dispensed from a polypropylene capillary (air-filled) inside a parallel plate capacitor. No tilting observed in the applied voltage range. (Distance between the plates of the capacitor: 2 cm; range of the applied voltage: 1-5 kV).
